# Functions of retinal astrocytes and Müller cells in mammalian myopia

**DOI:** 10.1186/s12886-022-02643-0

**Published:** 2022-11-24

**Authors:** Xuhong Zhang, Xin Yu, Yingying Wen, Le Jin, Liyue Zhang, Hong Zhu, Dongyan Zhang, Chen Xie, Dongyu Guo, Jianping Tong, Ye Shen

**Affiliations:** 1 Ophthalmology department, the First Affiliated Hospital of Zhejiang University, Qingchun Road No.79, Hangzhou, 310003 China; 2 Department of Ophthalmology, Shaoxing Central Hospital, Shaoxing, 312030 Zhejiang China

**Keywords:** Gene set enrichment analysis, Myopia, Astrocyte, Müller cell, Atropine

## Abstract

**Background:**

Changes in the retina and choroid blood vessels are regularly observed in myopia. However, if the retinal glial cells, which directly contact blood vessels, play a role in mammalian myopia is unknown. We aimed to explore the potential role and mechanism of retinal glial cells in form deprived myopia.

**Methods:**

We adapted the mice form-deprivation myopia model by covering the right eye and left the left eye open for control, measured the ocular structure with anterior segment optical coherence tomography, evaluated changes in the morphology and distribution of retinal glial cells by fluorescence staining and western blotting; we also searched the online GEO databases to obtain relative gene lists and confirmed them in the form-deprivation myopia mouse retina at mRNA and protein level.

**Results:**

Compared with the open eye, the ocular axial length (3.54 ± 0.006 mm v.s. 3.48 ± 0.004 mm, *p* = 0.027) and vitreous chamber depth (3.07 ± 0.005 mm v.s. 2.98 ± 0.006 mm, *p* = 0.007) in the covered eye became longer. Both glial fibrillary acidic protein and excitatory amino acid transporters 4 elevated. There were 12 common pathways in human myopia and anoxic astrocytes. The key proteins were also highly relevant to atropine target proteins. In mice, two common pathways were found in myopia and anoxic Müller cells. Seven main genes and four key proteins were significantly changed in the mice form-deprivation myopia retinas.

**Conclusion:**

Retinal astrocytes and Müller cells were activated in myopia. They may response to stimuli and secretory acting factors, and might be a valid target for atropine.

**Supplementary Information:**

The online version contains supplementary material available at 10.1186/s12886-022-02643-0.

## Background

Myopia, which can cause severe fundus oculi diseases, and even blindness [1], is becoming a global problem [2]. Numerous studies have uncovered many underlying mechanisms of myopia [3], including scleral expansion [4], retinal and choroid vessel changes during the process [5–7], oxidative stress [8] and hypoxia [9]. The decrease in vessel density and oxygen content causes the ocular hypoxia. Retinal glial cells are widely distributed and have many functions [10] in a variety of ocular diseases [11, 12]. Astrocytes are in close contact with blood vessels, and Müller cells provide radial support. Activated astrocytes can produce different mediators [13]. However, there are few reports of the relationship between retinal glial cells and myopia. Atropine is one of the most effective drugs used to delay myopia progression with the targets of the muscarinic receptor (MRs) and several non-MRs [14] as well as brain astrocytes [13]. We cannot exclude that atropine may benefit myopia by targeting glial cells.

In this study, we focused on the influence of retinal glial cells in the mice form-deprivation myopia (FDM) model. Through the immunohistochemical and bioinformatics analysis, we were determined to explore the function of retinal glial cells and the possible molecular mechanism.

## Materials & methods

### Animal and experimental procedure

All mice were obtained from Zhejiang Experimental Animal Center. They were raised in a 12/12 light/dark cycle and room temperature of 26℃. Food and water were provided ad libitum. Totally twenty-eight six-week male mice were used. After six days for FDM, their ocular structure was measured and then executed and their retinas were used for immunofluorescent staining, qPCR and western blot. All procedures were carried out in accordance with the National Institutes of Health Guidelines for the Care and Use of Laboratory Animals and with the ARRIVE guidelines. All procedures were approved by the Tab of Animal Experimental Ethical Inspection of the First Affiliated Hospital, College of Medicine, Zhejiang University (Approval No. 2021001).

### Mouse FDM

We followed the widely used method to induce FDM in C57BL/6 mice [15]. The right eyes of mice were covered with opaque packing tape for six days and the left eyes were left open (Fig. 2 A). In order to avoid scratching, we added a properly cut balloon around the head, neck and forelegs. All the procedures were performed under mild anesthetic with pentobarbitone (50 mg/kg intraperitoneal). All the used balloons were opaque white. We scattered the food on the ground and added water-rich AGAR gel besides water bottle to let them get food and water easily. The mice were checked daily to make sure the covers had not come off. In case the covers come off, we would exclude it and get a new mouse. The excluded mouse would be euthanasia by sevoflurane overdose.

### Ocular structure measurement

Ocular structure, including the ocular axial length (AL), central cornea thickness (CCT), anterior chamber depth (ACD) and vitreous chamber depth (VCD) (including the lens thickness), was measured using anterior segment optical coherence tomography (AS-OCT; SS-1000, Tomy Co., Japan) by a skilled operator. Each eye was measured three times, and clear images showing the pupils, lens boundaries and retina were captured (Fig. 2 B).

### Immunofluorescence staining in retina frozen sections and whole mounts

We performed this as previously described from our group [16]. Briefly, the mice were anesthetized with pentobarbitone (50 mg/kg, intraperitoneal), then myocardial perfusion was performed and the eyeballs were removed and fixed for two hours at 4℃. The mice corpses were disposed according to the practice of laboratory animal center of Zhejiang university. The ocular was excised and anatomized, and left the optic cup to be dehydrated. Then the optic cups were frozen with embedding medium (Neg-50; Thermo Scientific, Waltham, MA, USA). 15-μm-thick cryosections were collected and washed, dried, and blocked with confining liquid (10% normal donkey serum (NDS), 1% bovine serum albumin (BSA), and 0.3% triton X-100 in PBS) for one hour at room temperature in a humidity chamber. The primary antibody diluted with antibody diluent (1% NDS, 1% BSA, and 0.3% triton in PBS) and were incubated for one hour at room temperature. The secondary antibody was incubated for one hour at room temperature. DAPI (1:4000; C1002; Beyotime, Shanghai, China) was used to label the nuclei. The sections were mounted with 60% glycerinum under coverslips. For retina whole-mount retina, tissues didn’t need dehydration, the staining procedure was the same. Fluorescence images were acquired using a confocal microscope (FV1000; Olympus, Tokyo, Japan).

Primary antibodies used were anti-GFAP (1:300, 16,825–1-AP, Proteintech, Subsidiary in Wuhan, China); anti-EAAT4 (1:400, EAAT41-A, Alpha Diagnostic international, USA); secondary antibodies used were Alexa Fluor 488-conjugated Donkey anti-Rabbit IgG (1:1000, A-21206, Thermo Fisher Scientific); Alexa Fluor 546-conjugated Donkey anti-Rabbit IgG (1:1000, A-10040, Thermo Fisher Scientific).

### Bioinformatics analysis

**Fig. 1:**
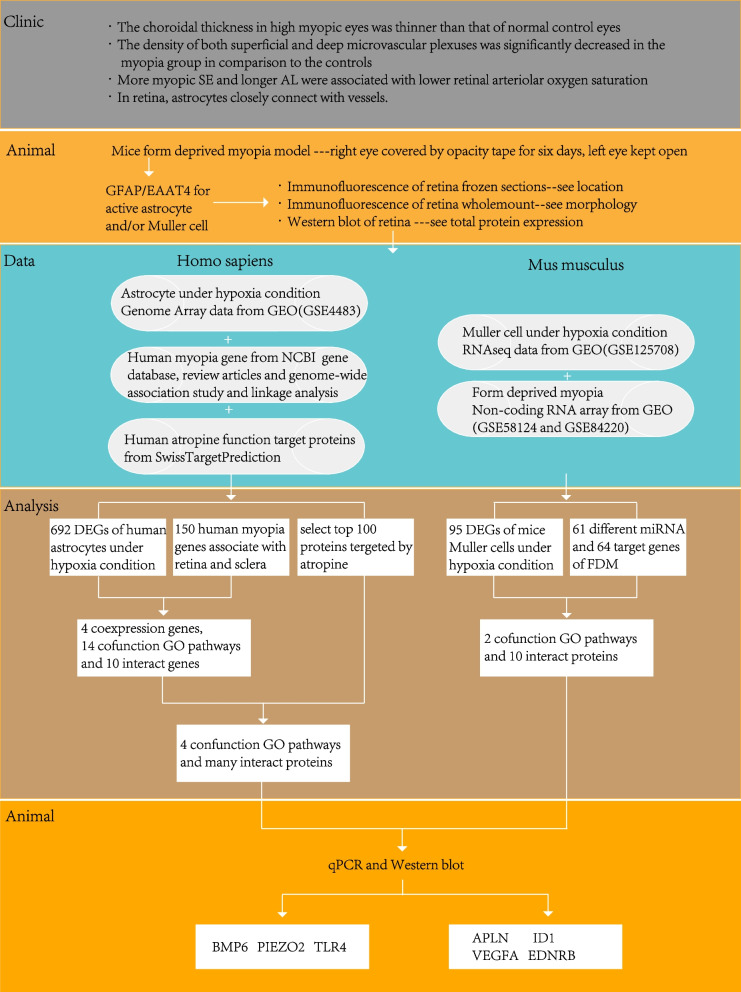
Flowchart of the experimental process

According to the previous clinic and experimental results, hypoxia of retina can play key role in myopia. So, we searched all the experiments related to hypoxia of glial cells in Gene Expression Omnibus (GEO) database. In their experiments, cultured cells were kept under 1% O_2_ for 24 h [17] and mice were raised in a normobaric, hypoxia chamber maintained at 7.5% O_2_ for either 48 h or 7 days [18]. Figure 1 presents the experimental flowchart. We downloaded the matrix files, or the results of online GEO2R analysis with the information file of the sequence platform from the GEO database. We then used R (version 3.6.3) for preprocessing. For the differential gene analyses, we identified differentially expressed genes (DEGs) based on a *p*-value less than 0.05 and fold change (absolute value of logFC) equal to or more than 2. To find the trend of total expression of the human myopia-related gene (HMRG) in astrocytes under hypoxia, we applied the analysis principle of Gene Set Enrichment Analysis (GSEA) to make an HMRG enrichment plot. We took the total 150 HMRGs as a reference group (Gene Ontology [GO] group), and found the astrocyte gene distribution and trend in this group. From GSE58124 and GSE84220, we found 61 differentially expressed miRNAs. miRNA does not usually encode proteins, so we could not directly perform functional enrichment analysis. Instead, we analyzed their functions through their corresponding mRNA using the online tool miRpath [19], which also provides the results of target genes. For genes, we performed GO analysis using another online tool, Metascape [20]. With its additional protein–protein interaction (PPI) results, we found the specific proteins and their functions in the associated pathways. By combining functional protein clusters using a third online tool STRING, we determined if the protein clusters interact with each other. The gene sequence GSE4483 was directly analyzed using Metascape. With the genes related to myopia, hypoxic glial cells, and atropine target proteins, we analyzed the contribution of atropine to myopia-related glial cells.

### Quantitative real-time PCR (qRT-PCR)

Total RNA was extracted using an Trizol reagent (Takara, Japan). A measure of 1000 ng of RNA from each sample was converted to cDNA using a Reverse Transcription kit (Takara, Japan). The cDNA was diluted 1:5 for subsequent qRT-PCR, which was conducted in 96-well plates with a QuantStudio 5 Real-Time PCR System (ABI, USA) and each sample was triplicated. Each reaction contained 0.4 µL primer (Tsingke Biotechnology, Beijing, China), 1 µL cDNA, 5 µL SYBR 2X Master Mix (Vazyme, Nanjing, China) and 3.6 µL DEPC-Treated Water and the amplification conditions was preincubation at 95 °C for 30 s, followed by 40 cycles of 95 °C for 5 s and 60 °C for 30 s (single acquire), and melting at 95 °C for 15 s, 60 °C for 1 min and 95 °C for 15 s. The relative expression of candidate genes was obtained using the comparative threshold cycle (2 − ΔΔCt) method [21].

Primers used were: *Id1*-F: CCTAGCTGTTCGCTGAAGGC; *Id1*-R: CTCCGACAGACCAAGTACCAC; *Bmp6*-F: AACGCCCTGTCCAATGACG; *Bmp6*-R: ACTCTTGCGGTTCAAGGAGTG; *Vegfα*-F: GCACATAGAGAGAATGAGCTTCC; *Vegfα*-R: CTCCGCTCTGAACAAGGCT; *Piezo2*-F: TACTATGCAAGGTTGTTTGGG; *Piezo2*-R: GTCTGGGTTTACTATGATCTTCC; *Tlr4*-F: ATGGCATGGCTTACACCACC; *Tlr4*-R: GAGGCCAATTTTGTCTCCACA; *Apln*-F: GAACTTCGAGGACTGGACC; *Apln*-R: CCTTCAATCCTGCTTTAGAAAGG; *Ednrb*-F: AAGCCACGCTGTCACTTCTC; *Ednrb*-R: GAGGAACGCATCAGACTGGA.

### Western blotting

Retinas were harvested after the animals were killed by pentobarbitone overdose. Retinas were homogenized in RIPA buffer that contained 1% Triton X-100, 0.1% SDS, 150 mM NaCl, 2 mM EDTA, 50 mM NaF, 10 mM sodium pyrophosphate, 1.0 mM Na_3_VO_4_, 1.0 mM PMSF, and complete protease inhibitor cocktail (Roche). The protein concentration in each sample was determined by a BCA protein assay (Bio-Rad Laboratories). Total protein in the samples was electrophoresed on 10% SDS-PAGE and transferred to polyvinylidene fluoride (PVDF) membranes (Millipore, USA). Primary antibodies were incubated overnight on a rocker at 4℃, followed by an appropriate secondary antibody conjugated to horseradish peroxidase (HRP) Signals were visualized using ECL-Plus Western blotting detection reagents (Thermo Fisher Scientific 1,863,096 and 1,863,097). All experiments were repeated three times.

Primary antibodies used were rabbit anti-GFAP (1:1000, 16,825–1-AP, Proteintech, subsidiary in Wuhan, China); anti-EAAT4 (1:3000, EAAT41-A, Alpha Diagnostic international, USA); anti-ID1 (1:500,18,475–1-AP, Proteintech, subsidiary in Wuhan, China); anti-EDNRB (1:500, 20,964–1-AP, Proteintech, subsidiary in Wuhan, China); anti-BMP6 (1:1000, A4538, ABclonal, China); anti-PIEZO2 (1:500, NBP1-78,624, Novus Biologicals, Littleton, CO, USA); anti-GAP (1:5000, 5174S, Cell Signaling Technology, USA).

## Statistical analysis

Images were quantified using ImageJ software (Fiji). GraphPad Prism 8 (GraphPad Software, Inc.) was used for statistical analyses and graphing. Significance is reported as *p* < 0.05, and data were expressed as mean ± SEM. Unpaired *t*-test or Mann–Whitney test were used to determine the significance level. Panels were put into multipart figures with Adobe Illustrator CC 2018. Experiments and analysis were performed by different people.

## Results

### Ocular structure after form deprivation

**Fig. 2:**
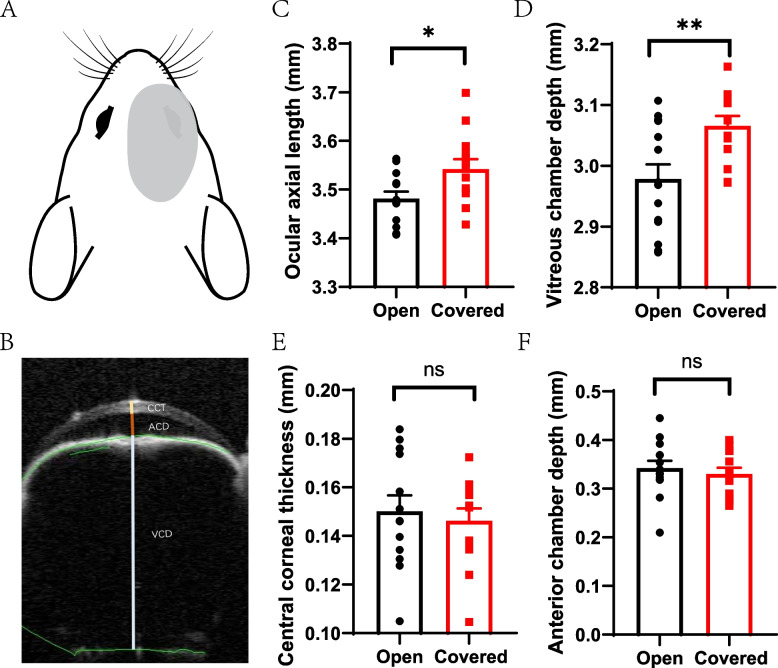
Mice were induced as form deprived myopia. **A** Diagrammatic drawing that the right eye was covered whereas the left eye was left open of every mouse. **B** Measurement of the ocular structure. CCT referred to central cornea thickness, ACD referred to anterior chamber depth and VCD referred to vitreous chamber depth. The axial length was the sum of CCT, ACD and VCD. Ocular axial length (**C**), vitreous chamber depth (**D**), central cornea thickness (**E**) and anterior chamber depth (**F**) were compared between the open and the covered eye. *t*-test, *n* = 14, * *p* < 0.05

After six days of form deprivation, the ocular AL (3.54 ± 0.006 mm v.s. 3.48 ± 0.004 mm, *t*-test, *p* = 0.027, *n* = 14) and VCD (3.07 ± 0.005 mm v.s. 2.98 ± 0.006 mm, *t*-test, *p* = 0.007, *n* = 14) of the covered eye were significantly elongated (Fig. 2 C and D). CCT (0.15 ± 0.001 mm v.s. 0.15 ± 0.002 mm, *t*-test, *p* = 0.64, *n* = 14) and ACD (0.33 ± 0.004 mm v.s. 0.34 ± 0.004 mm, *t*-test, *p* = 0.56, *n* = 14) were not changed (Fig. 2 E and F).

### Astrocytes and Müller cells in FDM mice retina

**Fig. 3:**
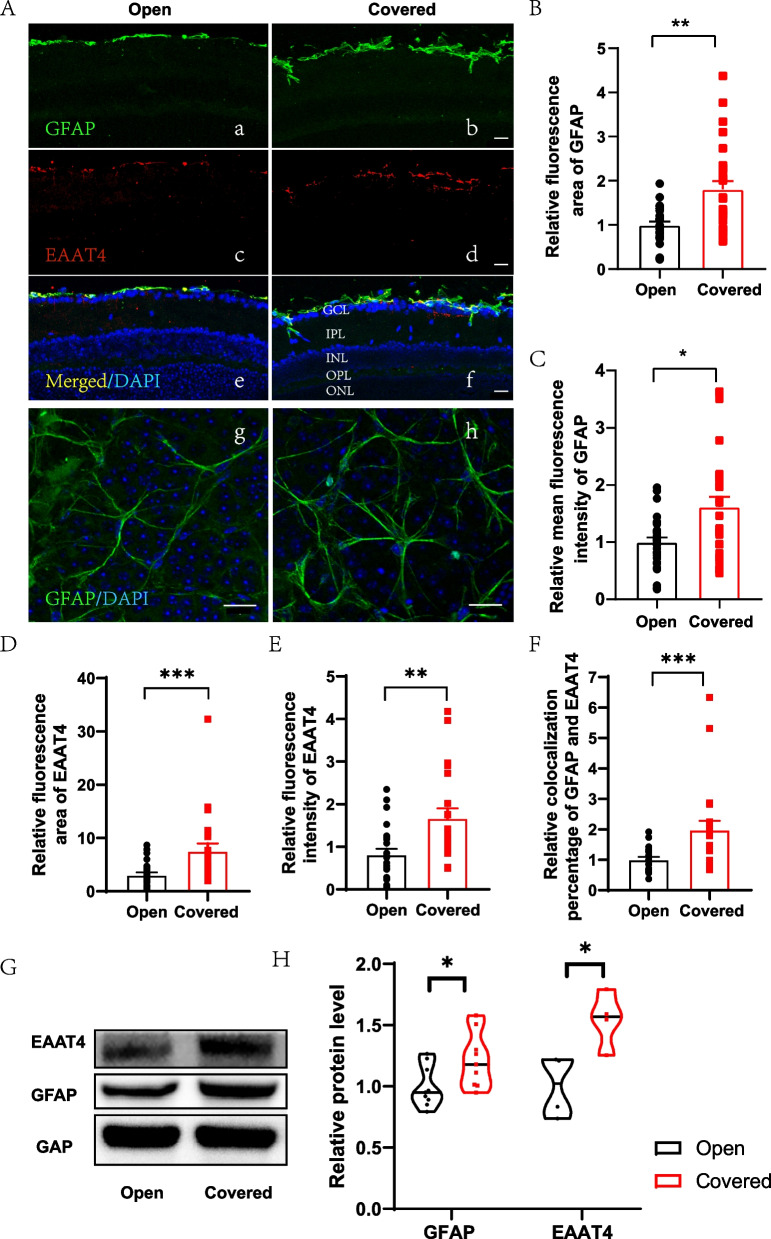
The expression of GFAP and EAAT4 elevated in FDM mice. **A** GFAP (green) and EAAT4 (red) staining in retinal section (**a**-**f**) and whole-mount (**g**-**h**). Relative fluorescence area of GFAP (**B**) and EAAT4 (**D**), relative mean fluorescence intensity of GFAP (**C**) and EAAT4 (**E**), relative colocalization percentage of GFAP and EAAT4 (**F**) were compared between the open and the covered eye. *n* = 12. (**G**-**H**) GFAP and EAAT4 expression determined by WB (the cropped images), *n* = 4–8. *t*-test or Mann–Whitney test, * *p* < 0.05, ** *p* < 0.01, ***p < 0.001

For GFAP, both the fluorescence area (Mann–Whitney test, *p* = 0.0013, *n* = 12) and intensity (Mann–Whitney test, *p* = 0.012, *n* = 12) were significantly elevated in the covered eye retina compared with the open eye (Fig. 3 Aa, Ab, Ag, Ah, B and C). In the open-eye retina, GFAP was on the inner side of the retinal ganglion cells (RGCs), while in the covered eye, the horizontal area was larger and displayed some thin fibers deep into the inner plexiform layer (IPL), which was much thicker than that of the open eye. In whole-mount retina, the morphology of GFAP was more star-like, and their connection with retinal vessels was obvious. However, in the open eye, GFAP was not as clearly visible and cell morphology was not distinct (Fig. 2 Ag and Ah). For EAAT4, also significantly increased fluorescence area (Mann–Whitney test, *p* = 0.0009, *n* = 6) and intensity (Mann–Whitney test, *p* = 0.0025, *n* = 6) were observed (Fig. 2 D and E). The colocalization percentage of GFAP and EAAT4 was also significantly higher in the retina of covered eye (Mann–Whitney test, *p* = 0.0004, *n* = 6, Fig. 2 F). Both GFAP and EAAT4 were increased in the covered eye determined by WB (*t*-test, *p* = 0.0226, Mann–Whitney test, *p* = 0.0286, respectively, *n* = 4–8, Fig. 2 G and H).

### Myopia-related and hypoxic astrocyte-related genes in human

A total of 217 human myopia genes are listed in the NCBI gene database, and 172 genes are listed in the NHGRI GWAS, Catalogue and ClinVar. The genes were systematically grouped by Tedja et al. [22] according to expression position. We selected all retina-related genes together with 27 pathologic myopia-related genes listed by Wu et al. [9]. We took hypoxic astrocyte-related DEGs from the GEO database (GSE4483). In total, we obtained 692 genes for hypoxic astrocytes and 150 genes for human myopia.

**Fig. 4:**
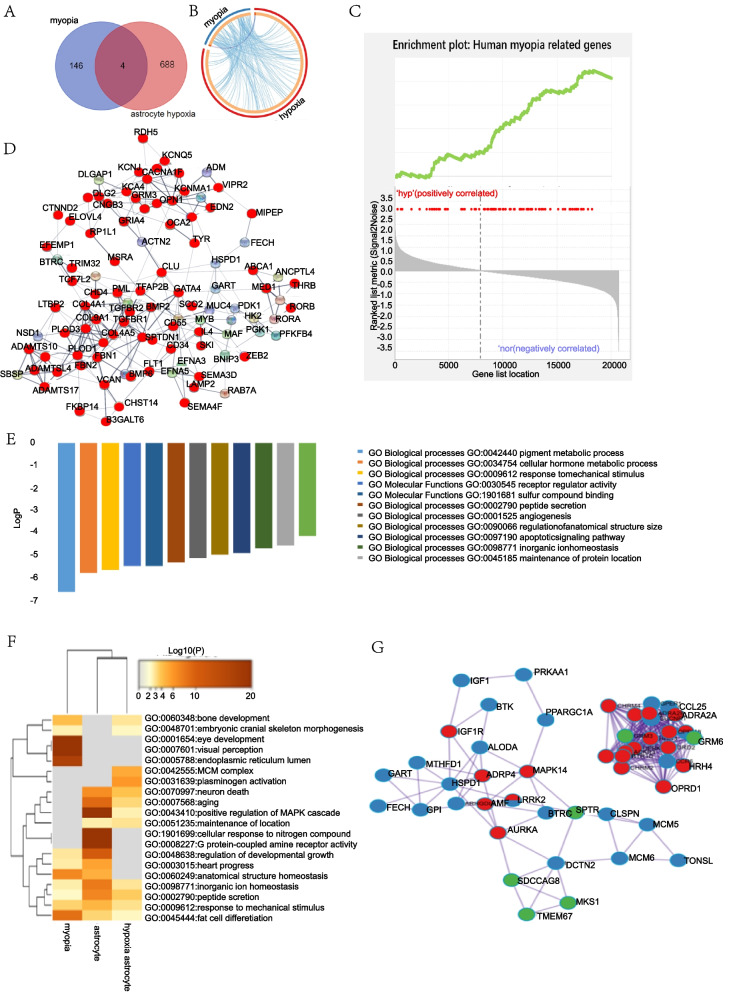
Myopia-related and hypoxic astrocyte-related genes in human. Venn diagram (**A**) and chordal graph (**B**) as well as GSEA analysis (**C**) shows the distribution and expression trend of myopia and hypoxia astrocyte genes. In partial PPI analysis (**D**), red nodes represent HMRGs, and thicker lines between nodes indicate a stronger relationship. (**E**) GO pathway analysis showing common pathways in the top 12 clusters. GO analysis of the common pathways (**F**), and PPI analysis of the connected nodes (**G**) (red: atropine target proteins, blue: myopia-related proteins, green: astrocyte-related proteins) show effect of atropine on myopia and astrocytes

Four genes were shared between the two groups of the Venn diagram (Fig. 4 A): *GRIA4, RP2, CNGB3*, and *ADAMTS10*. *GRIA4* is expressed in the cone ON bipolar cells and is responsible for the common refractive error. *RP2* and *CNGB3* are expressed in cones and rods and are associated with syndromic myopia. *ADAMTS10* is expressed in the sclera. Furthermore, the expression of all these genes increased when astrocytes suffered from hypoxia. As shown in Fig. 4 B, although the two groups covered all genes, the interaction between the groups was not uniform. By applying the analysis principle of the GSEA, we found that most HMRG enrichment was in the non-hypoxic part, indicating that most HMRG expression decreased when astrocytes suffered from hypoxia (Fig. 4 C). The PPI analysis revealed a strong connection of the HMRGs (red nodes) with hypoxic astrocyte-related genes (Fig. 4 D). Genes expressed in astrocytes, such as *ADM, ACTM2, FECH, EFNA3, EFNA5, MYB, MAF, ERBB2, RORA*, and *PPARGC1A*, also showed strong connections with HMRGs.

### Gene pathway analysis in human myopia and hypoxic astrocytes

To further explore the connection between myopia and astrocytes, we derived the main GO cluster pathways by analyzing the genes of the two groups (Fig. 4 E). The top 12 identified genes were involved in response to mechanical stimuli, peptide secretion, pigment metabolism, cellular and hormone metabolic processes, receptor regulation, activity, sulphur compound binding, angiogenesis, regulation of anatomical structure and size, apoptotic signaling pathway, inorganic ion homeostasis, maintenance of protein localization, and embryonic cranial skeleton morphogenesis.

### Atropine targets proteins with roles in myopia and astrocyte function

We collected a list of target proteins from SwissTargetPrediction and chose the top 100 as the most relevant. By comparing the gene lists for myopia, astrocytes, and atropine, we found some pathways that were common in any two of the selected lists, and some pathways even common in all three lists. Examples included pathways involved in inorganic ion homeostasis, peptide secretion, response to mechanical stimuli, and adipocyte differentiation (Fig. 4 F). Related genes including *ENG, PIEZO2, BMP6, KCNJ2, TLR4, IGF1R, THBS1, HIF1a, GRIA4, CNGB3, VIP, DRD2* and *TRPV1*. Excluding myopia, atropine had four GO clusters related to astrocytes. These clusters included neuronal death, ageing, positive regulation of the MAPK cascade, and maintenance of protein localization. The protein PPI analysis revealed the protein functions in clusters (Fig. 4 G). In our mice FDM model, we test the expression of related genes in the key pathway co-worked in myopia, astrocyte hypoxia and atropine targets, we found that *Bmp6*, *Tlr4* and *Piezo2* were all significantly changed at mRNA level (Mann–Whitney test, *p* = 0.0047; Mann–Whitney test, *p* = 0.0159; Mann–Whitney test, *p* = 0.0303, respectively. *n* = 4–8, Fig. 6 A), BMP6 and PIEZO2 were also significantly influenced at protein level (Mann–Whitney test, *p* = 0.0286, *t*-test, *p* = 0.0347, respectively. *n* = 4–8, Fig. 6 B and C).

### Myopia-related and hypoxic Müller cell-related genes in mice

**Fig. 5:**
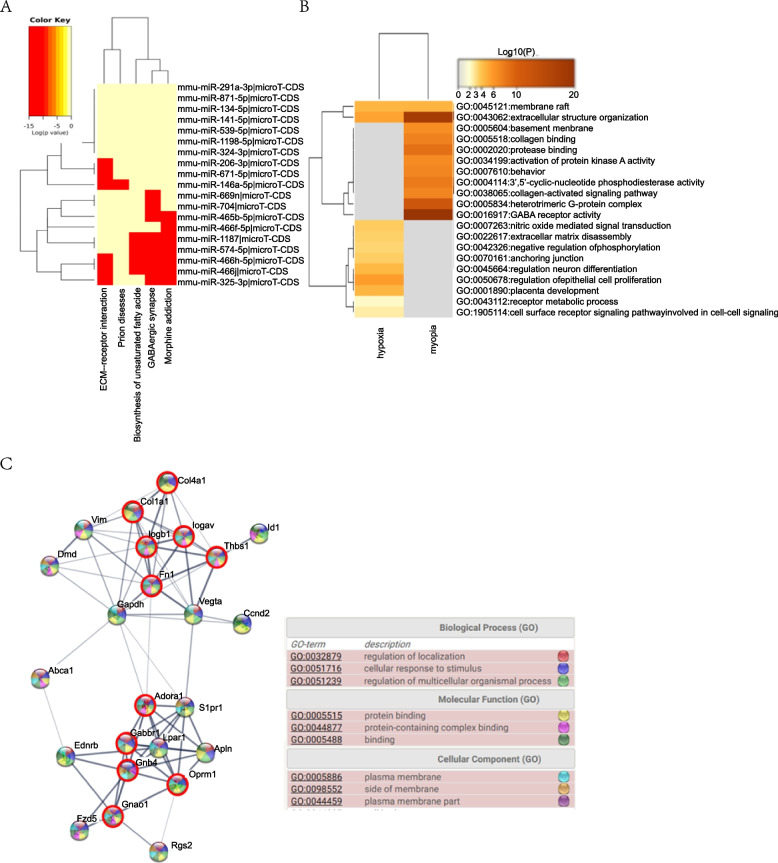
Myopia-related and hypoxic Müller cell-related genes in mice. **A** miRNA clusters in FDM mice, **B** the common pathways of the two conditions, and **C** PPI analysis and GO annotation results (nodes with a red circle represent myopia-related genes)

**Fig. 6:**
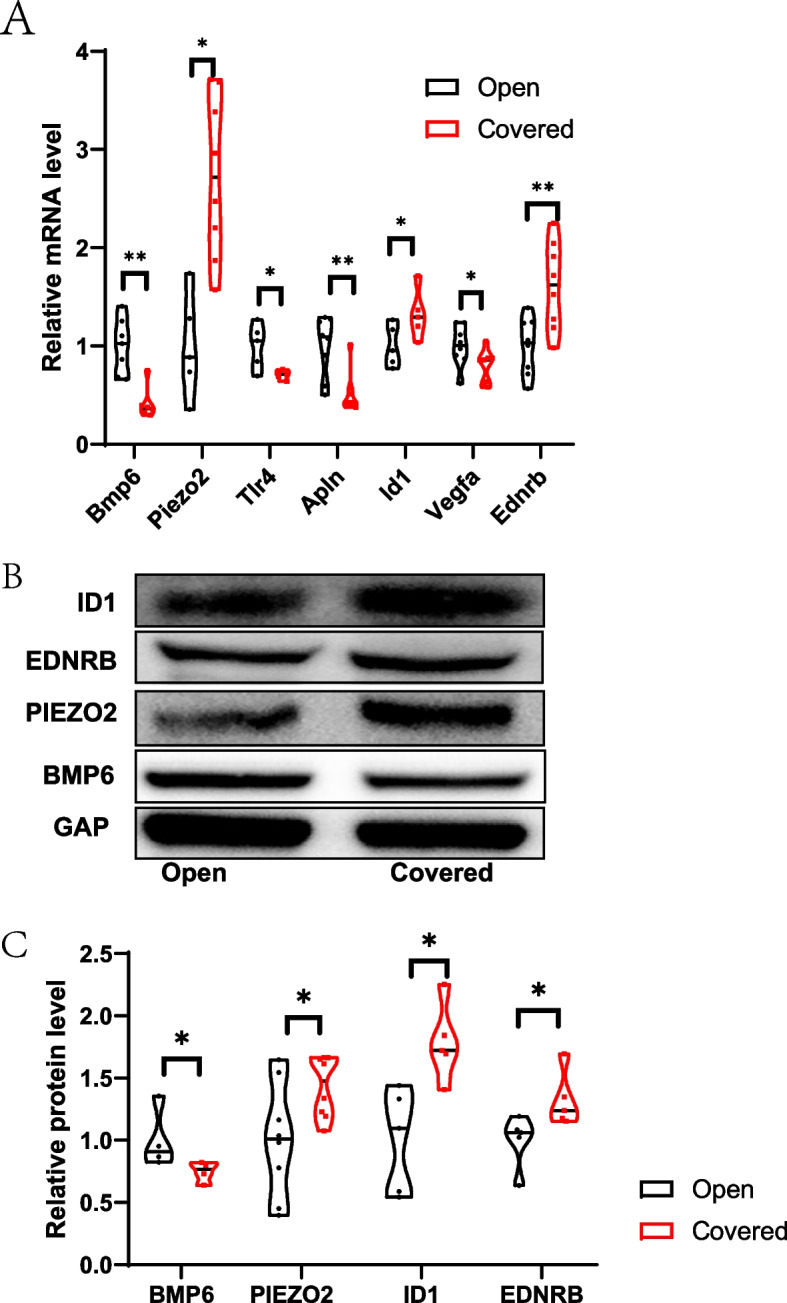
Expression of key genes as determined by qPCR (**A**) and WB (**B** and **C**) (the cropped images). *n* = 4–8. *t*-test or Mann–Whitney test, * *p* < 0.05, ** *p* < 0.01

We combined two similar databases, for which the primary data were miRNA expression in FDM mice. After analysis with miRpath, we generated a heat map of 33 miRNAs by Kyoto Encyclopedia of Genes and Genomes (KEGG) analysis. The main different miRNAs were mmu-miR-1187, mmu-miR-574-5p, mmu-miR-466hj, mmu-miR-325-3p, mmu-miR-465b-5p, and mmu-miR-465f-5p (Fig. 5 A). Based on the differentially expressed miRNAs, we identified the target genes in FDM mice. The five most significant pathways were GABAergic synapse, extracellular structure organization, heterotrimeric G-protein complex, protease binding, and 3’,5’-cyclic nucleotide phosphodiesterase activity. With the supplemental materials from the GEO database (GSE125708), we identified the target genes in hypoxic Müller cells. Both myopia and hypoxia in mice have relatively centralized function genes and proteins with few interactions in between. Membrane raft and extracellular structure organization were two GO cluster pathways common between the two groups (Fig. 5 B). The genes *Lpar1, S1pr1, Ednrb, Rgs2, Fzd5, Apln, Gapdh, Vim, Id1,* and *Vegfa* expressed by Müller cells code for proteins that link the two main functions (Fig. 5 C). They function mainly in the cytoplasm where they bind to proteins and have roles in the regulation of localization, cellular response to stimuli, and regulation of multicellular organismal processes. We also test the key genes in our mice FDM model, and found that *Apln*, *Id1, Vegfa* and *Ednrb* were all significantly affected at mRNA level (Mann–Whitney test, *p* = 0.0317; Mann–Whitney test, *p* = 0.003; *t*-test, *p* = 0.0446; *t*-test, *p* = 0.0057, respectively. *n* = 4–8, Fig. 6 A), ID1 and EDNRB were both significantly increased at protein level (Mann–Whitney test, *p* = 0.0159, Mann–Whitney test, *p* = 0.0317, respectively. *n* = 4–8, Fig. 6 B and C).

## Discussion

Ocular vessels have long been studied in myopia [5, 6, 23, 24]. Studies have reported that choroid thickness [7] as well as the density of retinal vessels [5] tends to decrease in myopia [25]. In our retina whole-mount GFAP photofluorogram, we also observed vessel change distinguished by astrocyte termini. A study that focused on the sclera concluded that the activation of the scleral hypoxia pathway causes myopia [9]. From the perspective of ocular blood supply, there is a close relationship between the retina and scleral hypoxia. The two important vessels that supply the inner retina and the outer retina-choroid-sclera are the central artery of the retina and the short posterior ciliary artery, respectively. They emerge directly from the ophthalmic artery. When the retina oxygen consumption increases, the short posterior ciliary artery supplies more oxygen to the outer retina and consequently less to the sclera. Many studies did determine retina oxygen consumption increases, such as photoreceptor differentiation [26] or synaptic transmission [27]. These findings suggest that in responding to specific visual signals, the outer retina photoreceptors are remodeled and consume more oxygen to generate energy, leading to hypoxia in the retina and sclera. This stimulates the retinal glial cells to release factors to the outer ocular layer. These factors together with the scleral factors cause final scleral remodeling. Our results also support the hypoxia function in myopia through many different pathways. In summary, ocular hypoxia may play an essential role in myopia.

Ocular enlarge and tissue stretch can also contribute to retina hypoxia. Retinal glial cells are expected to have an important role in retina signal transduction [28] and can be activated by a wide range of stimulus, including injury, inflammation and ischemia [29]. Stretching of the retina is thought to be a myopia risk factor during accommodation. Müller cells can secrete stretch-time-dependent factors [30]. Müller cells may also regulate the vitreous and retina fluid balance in myopia by expressing AQP4 in the end feet in chick FDM [31, 32]. Furthermore, Müller cells were found to release different factors depending on the extent of hypoxia. In mild hypoxia, Müller cells synthesized a protein factor that downregulated the expression of pigment epithelium derived factor (PEDF). However, under severe (or chronic) hypoxia, PEDF exhibited neurotrophic effects [33]. For astrocytes, Lin et al. [34] reported that the loss of astrocytes, which were co-localized with capillaries across the retina, led to the decrease in capillary density. Astrocytes also produce a wide range of factors targeting the RGCs [35, 36]. It is possible that in most experimental myopia models, young animals experience myopia during visual neurodevelopment, and may also have an visual acuity impact [37], in which astrocytes may play a role. In our mice FDM retinas, both astrocytes and Müller cells were significantly activated, indicated by protein expression and morphology change. Therefore, retinal glial cells may have an important role in myopia. Müller cells tend to be activated in response to large area stimuli such as ocular stretch and ocular fluid regulation, and thus transfer vision signals to the outer ocular layers, while astrocytes tend to transfer vision signals to higher vision centers by closely influencing RGCs and other neuronal cells.

**Fig. 7:**
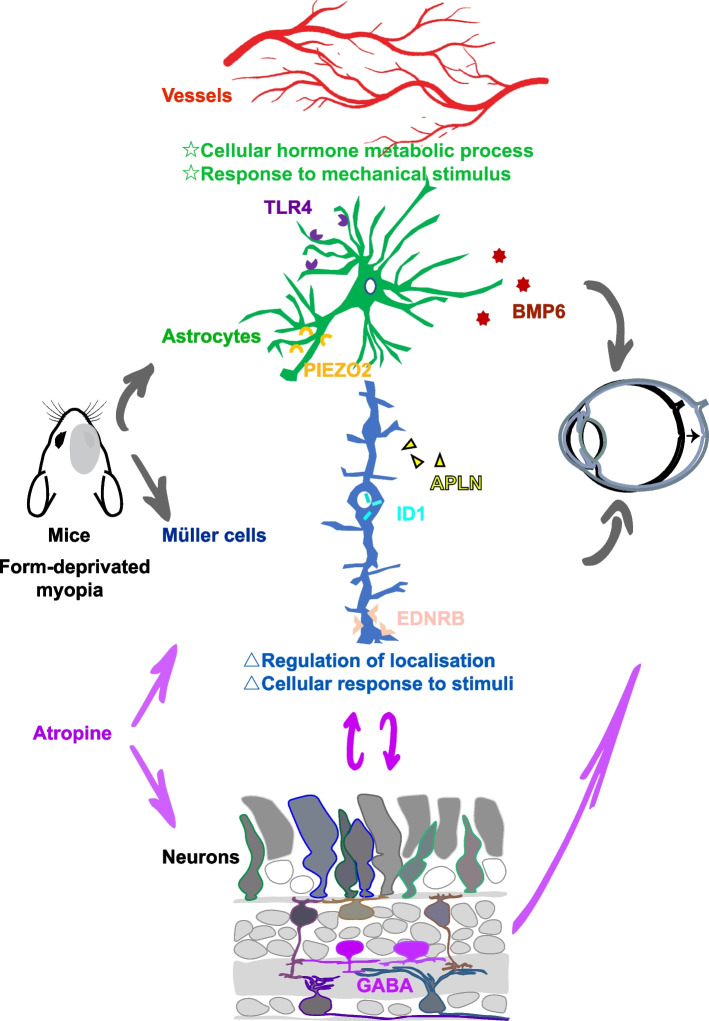
Schematic diagram of possible cellular and molecular mechanism in mice FDM. Astrocytes and Müller cells suffer from retina hypoxia, and work as an injury susceptor to regular the receptors or secreted proteins, thus work as a bridge to connect vessels and neurons. Finally, retinal signals transmitter to sclera and lead to the scleral remodeling and axial elongation in FDM. A possible mechanism for atropine treatment is that atropine targets on retinal neurons or glial cells and influence GABA release, which interplay with retinal glial cells, and finally protect FDM

From the bioinformatics analysis, we further identified 12 functional pathways in human astrocytes (Fig. 4 E) and two pathways in mouse Müller cells (Fig. 5 B). A previous study reported that cell proliferation activity and synaptic plasticity supersede other activities in the myopic retina [26], which made the retina becomes hypoxic and the functional pathways we list are activated to elicit a response to stretch and to regulate hormone and extracellular structure components. *Tlr4*, *Piezo2* and *Bmp6* were important in the activated pathways and we found that they were significantly changed in mice FDM (Fig. 6 A). Their functions in myopia or retina stretch have previously been reported. TLR4-dependent responses can be triggered when retina hypoxia and ischemia activated astrocytes and Müller cells [38]. Piezo channel as a mechanosensitive channel that senses pressure and shearing stress was found to be in astrocytes in the optic nerve head as well as the RGC layer of mice. The expression of retinal *Piezo2* was increased when suffering from retina stretch [39]. The increasing in our FDM retina indicated that retina under myopia was suffering from stretch. Atropine was used to inhibit the cholinergic activity, and can down-regulate BMP6 level [40]. *Bmp6* was also found to related with light induced ametropia [41]. The mechanism of atropine protect myopia might involve the GABAergic signaling by down-regulating GABA transporter 1 (GAT-1) [42], because GABAergic neurons have atropine-targeting muscarinic acetylcholine receptors (mAChRs). Atropine can also influence RGC possibly via the GABAergic pathway [43]. However, the GABA synthesis and release will be attenuated by TLR4 activation [44]. Inversely, activation of GABA receptor may also suppress the TLR4 signaling pathway [45]. In mice FDM retina the *Tlr4* was decreased, this may fail to down-regulate GABA and led to myopia. ID1 was the target of BMP, and it was found to have wide function on RGC protecting [46], ocular neovascularization [47] and Müller cells differentiation [48]. Its elevation in the FDM retina seemed to be a protective effect of feedback. EDNRB was an injury response susceptor of Müller cells [49], and it was sensitive for hyperglycaemia [50], glaucoma [51], and ischemic retinopathy [52]. The up-regulation of these factors implied that during FDM, the retina was in an injured condition. From an overall view of the differentially expressed factors listed above, we tended to point out the assumption that during FDM, the retina was under hypoxia condition and retina glia cells were activated. Their susceptor became more sensible and busy protecting blood vessels and neurons from damage. These actions seem to encompass the immediate response following the visual signal and neural reaction, which suggests that these pathways may be the crucial step leading to myopia, and the start of a harmful feedback loop that maintains ocular axis growth. Vessels-glia cells-neurons work together (Fig. 7), and among retinal neurons, amacrine cells plays an important role and may be a target for atropine by regulating GABA.

Many other evidences support our found about pathway function. Thyroid hormone signaling activity can decide the M/S-cone fate [53]. Matrix metalloproteinase 2 is a widely recognized extracellular remodeling factor that is generally found in the retina and scleral tissue in myopia [54–56]. Mechanical stimuli are common in the ocular system and have been proven to influence myopia in several studies [56, 57]. Atropine benefits myopia through MRs which induces mechanical changes [58]. Posterior eye growth was triggered in response to glucagon [59]. Insulin-like growth factor-1 [60] and vasoactive intestinal peptide also play a significant role in myopia [61–63]. Based on in silico results, we concluded that retinal glial cells can promote myopia by responding to mechanical stimuli, affecting hormone metabolism, peptide secretion, and cell differentiation.

### Conclusions

In summary, the main results we obtained from the in vivo and in silico experiments were that in myopia, retinal glial cells, namely, astrocytes and Müller cells, play a role under hypoxic conditions. The factors they secreted after stimulation may allow them to brought vessel and neurons together, and GABA might be a valid target for atropine.

## Supplementary Information

**Additional file 1** .

**Additional file 2** .

## Data Availability

The datasets analyzed during the current study are available in the Gene Expression Omnibus (GEO) database and searched the updated series for “myopia”, “astrocyte”, and “Müller”. We used four available series: GSE58124 (https://www.ncbi.nlm.nih.gov/geo/query/acc.cgi?acc=GSE58124) and GSE84220 (https://www.ncbi.nlm.nih.gov/geo/query/acc.cgi?acc = GSE84220) for mouse FDM, GSE125708 (https://www.ncbi.nlm.nih.gov/geo/query/acc.cgi?acc = GSE125708) for the mouse chronic hypoxia Müller cell gene, and GSE4483 (https://www.ncbi.nlm.nih.gov/geo/query/acc.cgi?acc = GSE4483) for human astrocyte function under hypoxia. For human myopia genes, we searched the National Center for Biotechnology Information (NCBI) gene database (https://www.ncbi.nlm.nih.gov/gene/?term=myopia), genome-wide association studies and linkage analyses [64, 65], as well as review articles. We also collected atropine functional target proteins using SwissTargetPrediction (http://swisstargetprediction.ch/).

## Abbreviations

MR: Muscarinic receptor
GFAP: Glial fibrillary acidic protein
FDM: Form-deprived myopia
PBS: Phosphate-buffered saline
NDS: Normal donkey serum
GEO: Gene Expression Omnibus
DEG: Differentially expressed gene
HMRG: Human myopia-related gene
GSEA: Gene Set Enrichment Analysis
GO: Gene Ontology
PPI: Protein–protein interaction
RGC: Retinal ganglion cell
IPL: Inner plexiform layer
KEGG: Kyoto Encyclopedia of Genes and Genomes
PEDF: Pigment epithelium derived factor
AL: Axial length
ACD: Anterior chamber depth
VCD: Vitreous chamber depth
CCT: Central corneal thickness
WB: Western blot
