# Supplementary figures and images for: Functions of retinal astrocytes and Müller cells in mammalian myopia
Source: BMC Ophthalmol. 2022 Nov 24;22:451. doi: 10.1186/s12886-022-02643-0 (PMC9686084; doi:10.1186/s12886-022-02643-0)

EAAT4

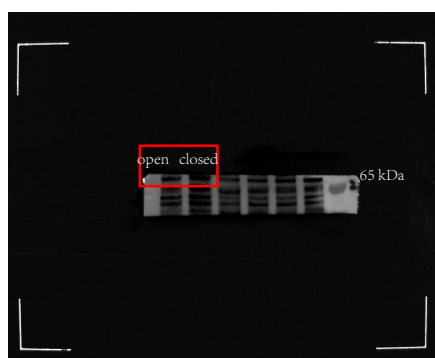

GFAP

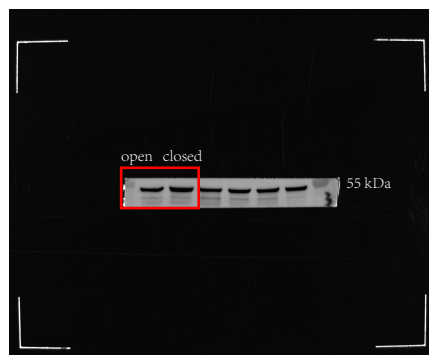

EAAT4

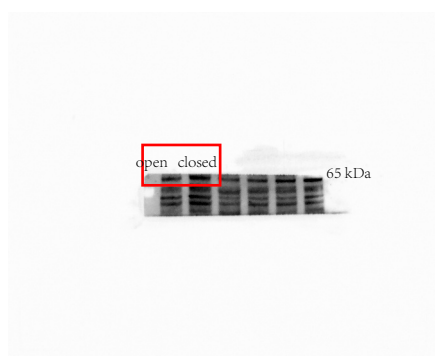

GFAP

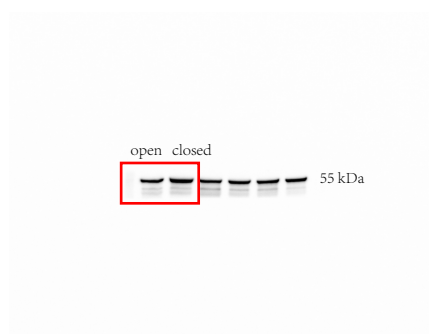

GAP

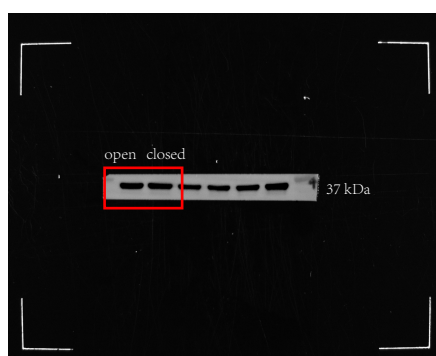

GAP

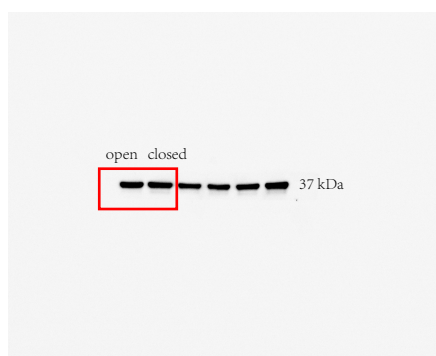

Supplement: Supplementary file 1 — Additional file 1 . [file 12886_2022_2643_MOESM1_ESM.pdf]

PIEZO2

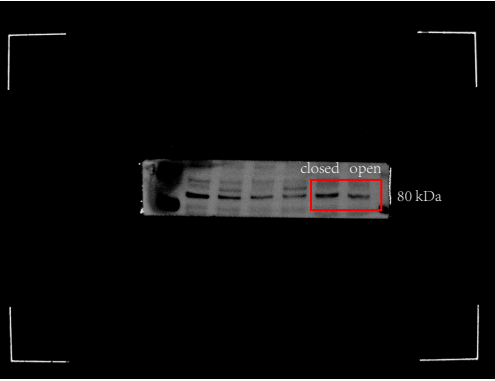

EDNRB

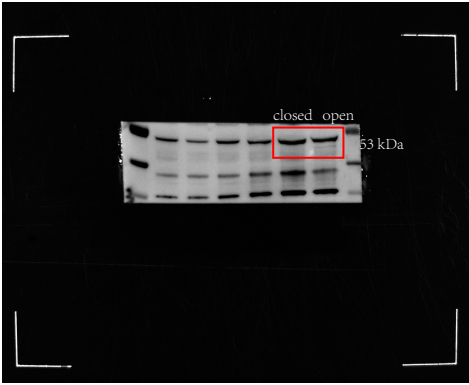

BMP6

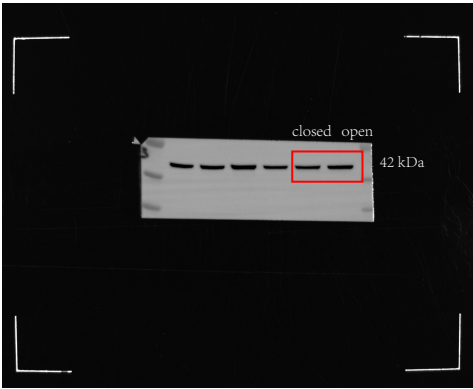

PIEZO2

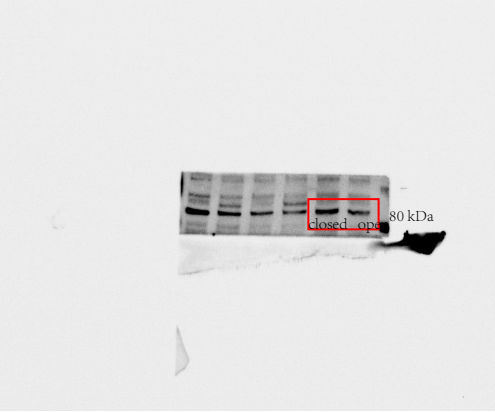

EDNRB

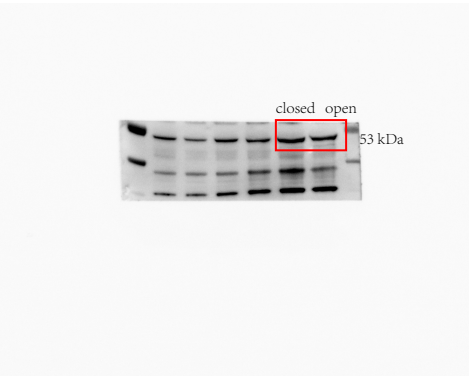

BMP6

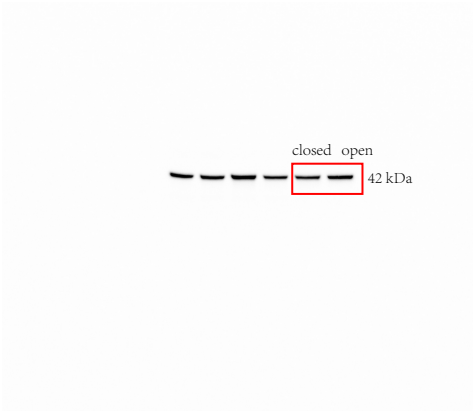

GAP

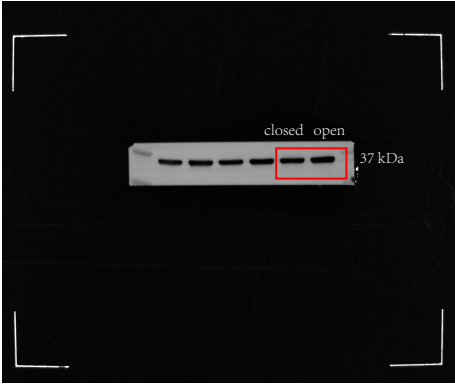

ID1

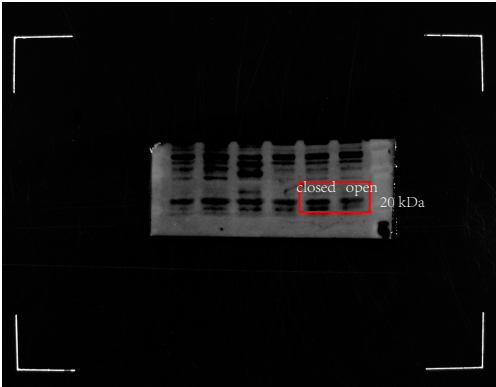

GAP

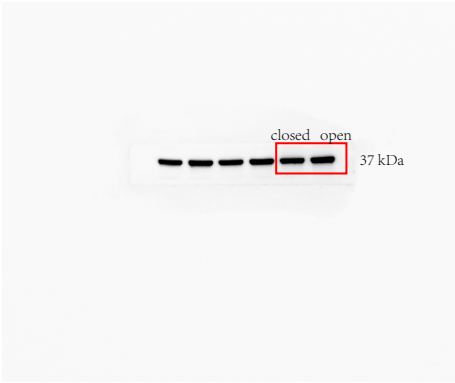

ID1

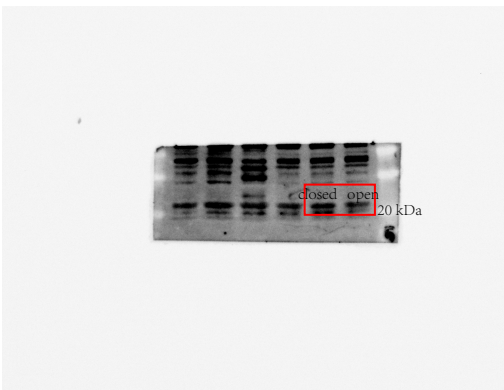

Supplement: Supplementary file 2 — Additional file 2 . [file 12886_2022_2643_MOESM2_ESM.pdf]
